# Motorized Treadmill and Optical Recording System for Gait Analysis of Grasshoppers

**DOI:** 10.3390/s21175953

**Published:** 2021-09-05

**Authors:** Leslie Barreto, Ahnsei Shon, Derrick Knox, Hojun Song, Hangue Park, Jeonghee Kim

**Affiliations:** 1 Department of Engineering Technology and Industrial Distribution, Texas A&M University, College Station, TX 77843, USA; leslie.barreto28@tamu.edu; 2 Department of Multidisciplinary Engineering, Texas A&M University, College Station, TX 77843, USA; ahnseishon@tamu.edu (A.S.); hangue.park@tamu.edu (H.P.); 3 Department of Electrical and Computer Engineering, Texas A&M University, College Station, TX 77843, USA; derrickknox@tamu.edu; 4 Department of Entomology, Texas A&M University, College Station, TX 77843, USA; hsong@tamu.edu

**Keywords:** closed-loop motor control, grasshoppers, insect gait analysis, motorized treadmill, optical recording system

## Abstract

(1) Background: Insects, which serve as model systems for many disciplines with their unique advantages, have not been extensively studied in gait research because of the lack of appropriate tools and insect models to properly study the insect gaits. (2) Methods: In this study, we present a gait analysis of grasshoppers with a closed-loop custom-designed motorized insect treadmill with an optical recording system for quantitative gait analysis. We used the eastern lubber grasshopper, a flightless and large-bodied species, as our insect model. Gait kinematics were recorded and analyzed by making three grasshoppers walk on the treadmill with various speeds from 0.1 to 1.5 m/s. (3) Results: Stance duty factor was measured as 70–95% and decreased as walking speed increased. As the walking speed increased, the number of contact legs decreased, and diagonal arrangement of contact was observed at walking speed of 1.1 cm/s. (4) Conclusions: This pilot study of gait analysis of grasshoppers using the custom-designed motorized insect treadmill with the optical recording system demonstrates the feasibility of quantitative, repeatable, and real-time insect gait analysis.

## 1. Introduction

Gait refers to a pattern of movement of the limbs during locomotion over a solid substrate. When an animal with limbs executes locomotion, a particular gait is selected to optimize stability and efficiency according to given environmental conditions and the objective of locomotion, in response to internal and external stimuli. As multiple muscles are involved in generating each kinematic output and a continuum of proper kinematic outputs constitutes the optimal gait [1,2], well-orchestrated neural activities are crucial for modulating the gait pattern in real time [3,4]. In this regard, gaits have been used as an important tool for understanding the operation of the complex neural circuitry [5,6]. In other words, gaits are a complex and harmonious outcome of the neural activities. With the complexity of the neural activities in gait formation, a minor change in the neural activities can result in significant changes in gait outcome [7]. Decades of gait studies in human and vertebrate animals have produced the two-level half center model of the locomotor central pattern generator (CPG), which represents the neural organization in the spinal cord [8,9]. Gait studies play an important role not only in understanding neural circuitry, but also in diagnosing neurological disorders and evaluating the rehabilitation process. For example, they have been used as a tool to diagnose Parkinson’s disease and ataxia [10] and to evaluate a rehabilitation process after spinal cord injury or stroke [11,12]. Gait studies have also advanced robotics, such as terrain mobile robots and tactical robots [13,14]. Gait pattern dependencies on genetic and environmental factors can also contribute to the study of biological evolution [15,16,17].

In order to properly study gaits, special equipment should be installed on the subject’s body and/or the surroundings in order to record kinematic and kinetic variables. In cases of an overground walking environment, for both human and animal subjects, wearable sensor units can record kinematic variables, and pressure mats or force plates can record kinetic variables [18,19]. However, data resolution of the gait analysis in overground walking is usually limited because of the limited number of wearable nodes and sensors covering a large space. The treadmill has been introduced in gait studies to overcome these limitations of data recording. The gait on the treadmill and the associated clinical outcomes can be repetitively recorded in a confined space [20], and an optical recording system with reflective markers can easily provide millimeter-scale high-resolution data [21,22,23,24]. Furthermore, a treadmill can provide multiple options of gait interventions, including speed change and inclination, which can be useful for studying the nervous system and its sensorimotor loop operation [25]. To take advantage of the treadmill setting, multiple variations of the treadmill have been developed, such as instrumented treadmill, split-belt treadmill, and body-weight supported treadmill [4,26]. An instrumented treadmill provides kinetic variables, such as ground reaction force; a split-belt treadmill allows for regulation of speed for each side of the body independently, which is often used to provide challenging conditions to the paretic side; and a body-weight supported treadmill provides support for the subjects under severely disabled conditions [27,28,29].

Until now, gaits have been mainly investigated in human or other vertebrate models, and for this reason, the treadmill and the gait recording system have also been mainly developed for these large-bodied subjects. Gait studies on insects have been relatively limited [30,31,32], although they provide multiple advantages over the vertebrate models. Insects have a relatively simple nervous system, which provides straightforward interpretation; surgical procedures on insects are relatively simple, the number of test subjects can be much larger than that of the vertebrate subjects, and experiments can be repeated multiple times in a short time period given their short life cycle. Insect treadmills have been developed for ants, cockroaches, and crickets using a rolling-ball type air-cushioned spherical nonmotorized treadmill without any real-time gait analysis [33,34,35]. However, this type of treadmill is not easily adoptable for large-bodied insects, such as grasshoppers and stick insects, because of the differences in body size, walking patterns and speed, and leg length. Large-bodied insects with clearly visible leg joints have several advantages over smaller insects for gait studies because the movement of each joint can be easily detected and quantified due to their large legs that are strong enough to locate reflective markers, which can provide detailed kinematic information [36]. Although most of the previous studies on insect gaits did not use insect treadmills, they nevertheless showed interesting gait patterns in terms of the effect of walking speed and sensory feedback. For example, cockroach gaits have been studied to investigate the mechanism of rapid terrestrial locomotion, in terms of the stepping pattern and interleg coordination. Cockroaches use a typical alternating tripod stepping pattern at a speed less than 1.0 m/s, but at a high speed over 1.0 m/s, they use a metachronal stepping pattern or sometimes use only two or four legs [37,38]. The gait of stick insects has been investigated to study the intersegmental influence between local CPG and the effect of sensory feedback. The results suggested that local sensory feedback could override the intersegmental influence [39,40]. The gait of locusts (grasshoppers capable of swarming) has also been investigated, in terms of the metastability of the double-tripod gait [41] and the strategy for walking on rough terrain [42]. The results suggest that locusts intermittently employ metastable walking gaits without adopting rigid gaits, but employ a wide range of stepping patterns when walking on rough terrains.

**Figure 1:**
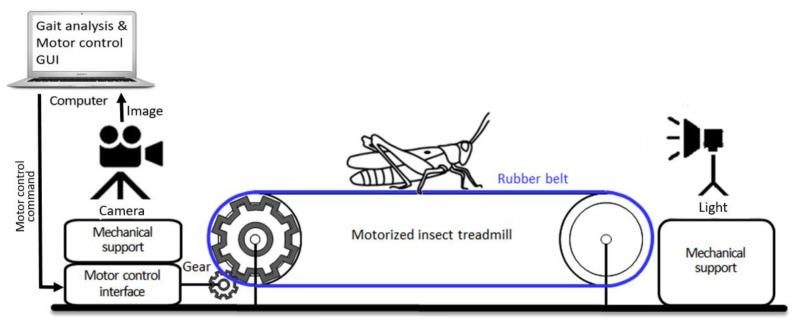
Overall experimental setup of the motorized insect treadmill, which is capable of changing belt speed, with optical recording system.

In the previous studies of insect gaits, gait changes according to the walking speed have not been investigated well, especially at slow to normal walking speeds, because of the limited capability of controlling the walking speed of insects and the limitations in real-time quantitative gait analysis. Indeed, it is difficult to force the insects to walk consistently at several different speeds. One cockroach study investigated the effect of walking speed on gaits, but only at a high walking speed by forcing them to run away from a threat [37]. In this study, we present a novel system for recording insect gaits with a wide range of walking speed, controlled by the belt speed, from slow to fast (i.e., 0.1–1.5 cm/s), using a customized treadmill built for a large-bodied grasshopper species and an optical recording system (Figure 1). This treadmill system is motorized with a closed-loop speed control capability and the graphical user interface (GUI) for controlling, and we demonstrate the development of treadmill electronics and software, the design of the mechanical system, and its utility with actual data collection with grasshoppers. We outline different types of novel gait parameters that can be generated using this new system.

## 2. Materials and Methods

### 2.1. Development of the Grasshopper Treadmill

#### 2.1.1. Treadmill Electronics

**Figure 2:**
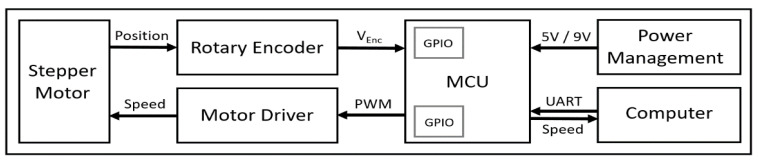
Overall block diagram of electrical system of the grasshopper treadmill.

We custom-developed electrical and mechanical components of our treadmill system, specifically designed for quantifying grasshopper gaits. The block diagram of the electrical system of the grasshopper treadmill is shown in Figure 2. The electrical system of the treadmill consisted of a microcontroller (MCU; ATmega328, Microchip, Chandler, AZ, USA), a dual-shaft stepper motor (SY42STH38-1684A, Changzhou Songyang Machinery & Electronics, Changzhou, China), a stepper motor driver (DRV8825, Texas Instruments, Dallas, TX, USA), a rotary encoder (KY-040, Handson Technology, Johor Bahru, Malaysia), 12 V AC adapter (UpBright, Shenzhen, China) to supply the motor, and two step-down voltage regulators (LM2596 and LM7805, Texas Instrument, USA) to provide 9 V to the microcontroller and 5 V to motor driver and encoder. The power management circuit converted wall power (120 V) to 12 V for the motor and 5 V for the rest of the system.

#### 2.1.2. Software

Due to the fine speed control of the treadmill, we selected the dual-shaft stepper motor with the rotary encoder to implement the feedback loop of the speed control. To make the closed-loop treadmill system, the actual speed of the treadmill was estimated from the position of the rotary encoder and was controlled/modified through a pulse width modulation (PWM) signal to fine-tune the speed of the treadmill. The controllable range of the belt speed was from 0.1 to 3.0 cm/s, and the speed could be adjusted on the LabVIEW software at a resolution of 0.01 cm/s. 

**Figure 3:**
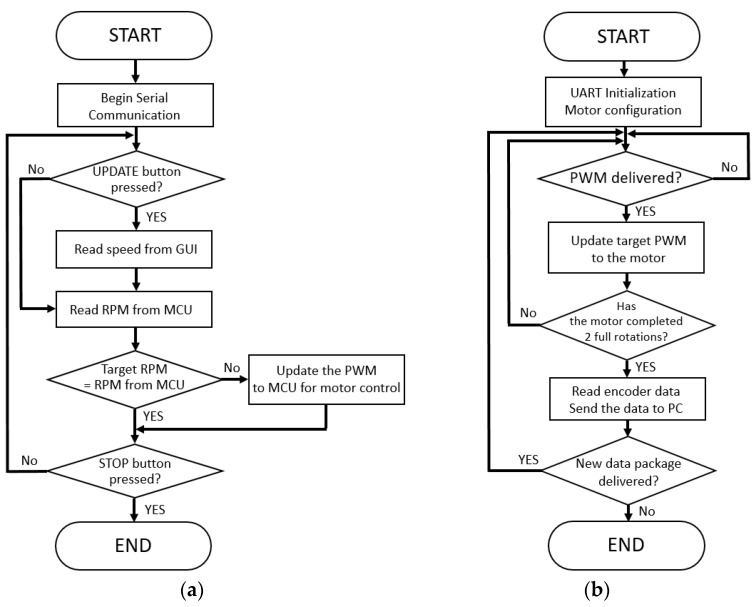
Software flowcharts of the (**a**) LabVIEW interface and (**b**) firmware of the microcontroller.

To implement the closed-loop speed control, we built two software loops for the GUI and the microcontroller, separately. The first loop works as shown in Figure 3a, and it is built on the LabVIEW program. Once the LabVIEW software initiated the serial communication with microcontroller, the target belt speed could be set via the GUI from a laptop computer. It first read the target speed of the treadmill that was selected by the users on the screen and converted the target speeds to the revolutions per minute (RPM) for the motor. Afterward, it read the current RPM from the microprocessor through the encoder and compared the target RPM with the actual RPM. If there was any difference between target and actual RPMs, then the difference was converted to the PWM and conveyed to the microcontroller for the stepper motor control.

The second loop works as shown in Figure 3b, and it was programmed on the microcontroller firmware. The microcontroller-controlled motor speed was based on the target speed as PWM that was delivered from the LabVIEW software via the UART interface. Once the motor completed two full rotations, then the microcontroller read the encoder to convey the current motor speed to the LabVIEW software. 

#### 2.1.3. Mechanical System

**Figure 4:**
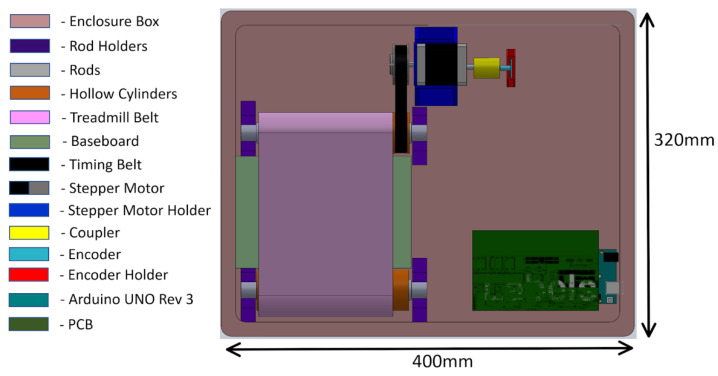
Mechanical design of the treadmill with enclosure.

**Figure 5:**
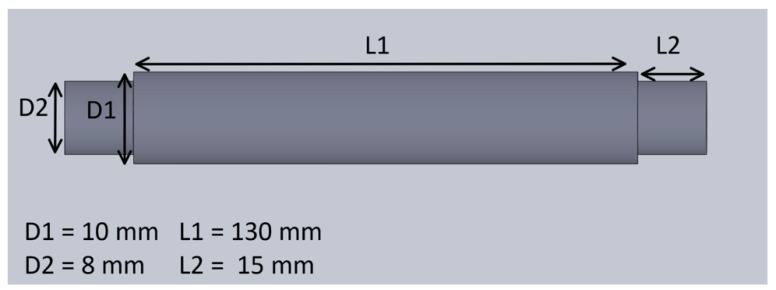
The dimensions of the hollow cylinders.

The mechanical system of the treadmill consisted of a pair of rollers, a timing gear, a timing belt, a treadmill belt, and a plastic enclosure. The mechanical design of the treadmill with enclosure is shown in Figure 4. The treadmill belt was made of rubber and steered by motorized rollers, which consisted of a rod, two bearings, and a hollow cylinder. The design and the dimension of the hollow cylinder are shown in Figure 5. The dimensions of the rollers were 30 mm in outer diameter and 200 mm in length. One of the rollers was integrated with the timing gear and a belt to motorize the treadmill. A GT2-type synchronous wheel, with 60 teeth with a bore diameter of 5 mm, worked as a timing gear. The timing belt (22 mm in length, 10 mm in width, and 2 mm in pitch) was made using rubber. The treadmill belt went around the rollers with enough tension to allow horizontal movement, and the dimensions of the treadmill belt on the top were 200 mm in length and 130 mm in width. The hollow cylinders and enclosures were designed in 3D Design software (Solidworks 2018, Solidworks, Waltham, MA, USA) and printed using a 3D printer (Da Vinci Mini W, XYZ printing, San Diego, CA, USA), with printing materials using carbon fiber, ABS, and PLA to provide durability and strength, as well as light weight. 

### 2.2. Optical Recording and Analysis of Grasshopper Gaits

**Figure 6:**
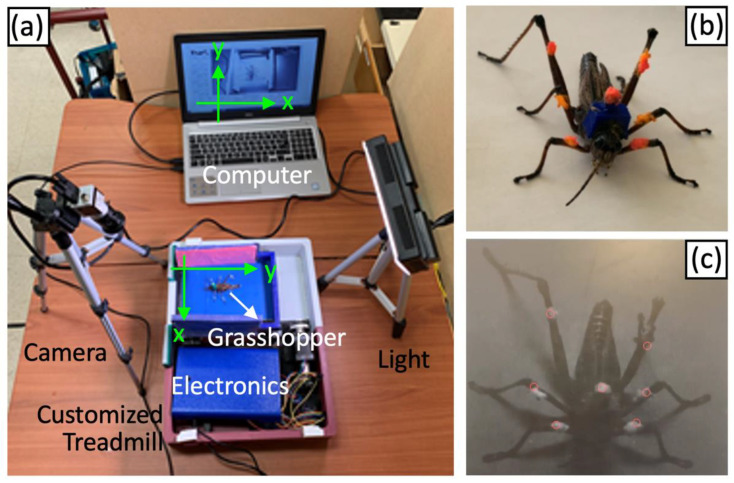
(**a**) Overall experimental setup of the motorized insect treadmill, which is capable of changing belt speed, with optical recording system. (**b**) One of the grasshoppers with the reflective markers (fluorescent pigment powder) and (**c**) the output of the offline Kanade–Lucas–Tomasi (KLT) algorithm using MATLAB.

The overall experimental setup of the motorized insect treadmill with optical recording system is shown in Figure 6a. We used reflective markers attached to each leg right above the knee joint to record grasshopper gaits (See Figure 6b). To make the reflective markers, we used fluorescent pigment powder mixed with gel-type super glue (Loctite Ultra Gel Control), and we applied the markers to each leg as a small dot (about 5 mm in diameter). High-intensity LED light (LED-7100T, Genaray, North Brunswick, NJ, USA) was turned on to increase the light reflection, and a Basler Aca1300-75gm camera recorded the walking with the right aperture to distinguish the reflective markers from the background. Note that the selected camera is capable of capturing 88 frames per second (with 1280 × 1024 pixel resolution), which means that each gait cycle can be captured more than 88 times with <11 ms resolution (<1% of the gait cycle). We used the LabVIEW software to read and save the image data from the camera and to provide the control of reflection ratios for each color to optimize the quality of recording for the specific-colored pigment. 

To track the seven reflective markers placed on the grasshopper subject (six for each leg and one for center of body), we utilized a point tracker object from the MATLAB signal processing package [43]. The point tracker object tracks a set of points using the Kanade–Lucas–Tomasi (KLT) algorithm [44,45,46], a feature tracking algorithm that is widely used for video stabilization, camera motion estimation, and object tracking. We first found corners using a minimum eigenvalue and then tracked a set of points using the KLT feature tracking. The KLT algorithm generated multiple sets of points that were close to each marker. We visually inspected the points that remained in the right position during the whole set of video files. We manually selected the closest point from each marker and generated the output for each marker (x and y position of the video screen). After extracting the x and y coordinates of each marker during the recorded file, we applied a low pass filter using 10th-order Butterworth with 1.8 Hz cutoff frequency. The cutoff frequency of the low pass filter was selected as 1.8 Hz, which was determined as twice the maximum gait frequency (0.9 Hz). The order of the filter was selected as 10th order, determined on a trial-and-error basis to maximize the performance. In this study, the analysis was completed offline after the recording of the images of the gait. An example output of the offline KLT algorithm using MATLAB is shown in Figure 6c.

In order to identify the gait phase (stance vs. swing), we focused on the change in the *y*-axis coordinate for each leg marker, where the *y*-axis is aligned with the moving direction of the treadmill belt. We first subtracted the *y*-axis coordinate of each leg marker from the *y*-axis coordinate of the body marker, which canceled the offset caused by any body movement. We then identified the direction of change in the *y*-axis coordinate of each leg marker. We categorized the points with the positive change in the *y*-axis coordinate (i.e., forward direction) as the swing phase and the points with the negative change (i.e., backward direction) as the stance phase. Note that when the leg was placed on the belt (i.e., stance phase), the corresponding leg would move in a caudal direction, and the leg would move in a rostral direction when the leg was in swing. We repeated the categorization for each of six legs. The gait cycle duration was defined as a sum of “stance” and “swing” phases individually for each leg.

### 2.3. Calculation of Walking Speed, Stance Duty Factor

Walking speed was calculated for each gait cycle, using the change in the *y*-axis coordinate of the body marker and the belt movement speed, as shown in the following equation:

(1) $$Walkingspeed=\frac{\Delta y_{bodymarker}}{Gaitcycleduration}+beltspeed$$

Stance duty factor was calculated for each gait cycle for each leg, using the stance phase duration and gait cycle duration, as shown in the following equation below:

(2) $$Stancedutyfactor=\frac{Stancephaseduration}{Gaitcycleduration}$$

### 2.4. Study Insects

We used the eastern lubber grasshopper, *Romalea microptera* (Orthoptera: Romaleidae) as test subjects. The grasshoppers used in our study were collected as nymphs from southern Florida in March 2019 and were brought to Texas; we reared them to adults in a large metal cage (61 cm × 61 cm × 61 cm) in a walk-in environmental chamber in the Department of Entomology at Texas A&M University. All of the grasshoppers were reared at 14 h light: 10 h dark, 30 °C, and an average of 50% humidity and fed daily on romaine lettuce and wheat bran. For this study, we used 3 male adults. The grasshoppers were starved for at least 8 h prior to the experiment. This particular species of grasshopper is a voracious eater and would eat frequently in bouts throughout the daytime (under light condition). The 8-h starvation is a sufficient time to allow for digestion and excretion, and therefore, the insects were motivated enough to forage for food. Before the experiment, we trained the grasshoppers to walk on the motorized insect treadmill with a food reward and an air flow. We allowed the grasshoppers to walk on the treadmill for several minutes before the experimental session, with several trials with different belt speeds ranging from 0.1 to 1.5 cm/s.

## 3. Results

### 3.1. Relationship between Stance Duty Factor and Walking Speed

**Figure 7:**
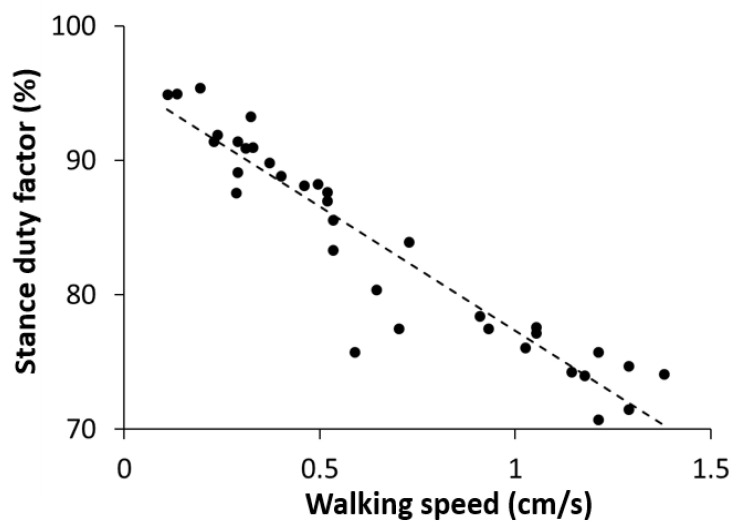
Measurement data representing the stance duty factor, averaged over all six legs, according to the walking speed. The diagonal dashed line is the least-squares regression line (R^2^ = 0.8895, *p* < 0.001).

A stance duty factor is defined as the percentage of the duration in which a given leg is on the ground over the whole gait cycle. We measured the stance duty factors for all six legs at different walking speeds and calculated the average over the six legs at each walking speed. We found that the stance duty factor was negatively correlated with the walking speed (Figure 7). The stance duty factor was 85–95% at a slow walking speed (0.1–0.55 cm/s), 75–85% at a medium speed (0.55–0.85 cm/s), and 70–80% at a fast speed (0.85–1.5 cm/s). The criteria for determining the degree of speed were based on a previous study on the locust walking speed [47].

### 3.2. Gait Phase and Interleg Coordination

**Figure 8:**
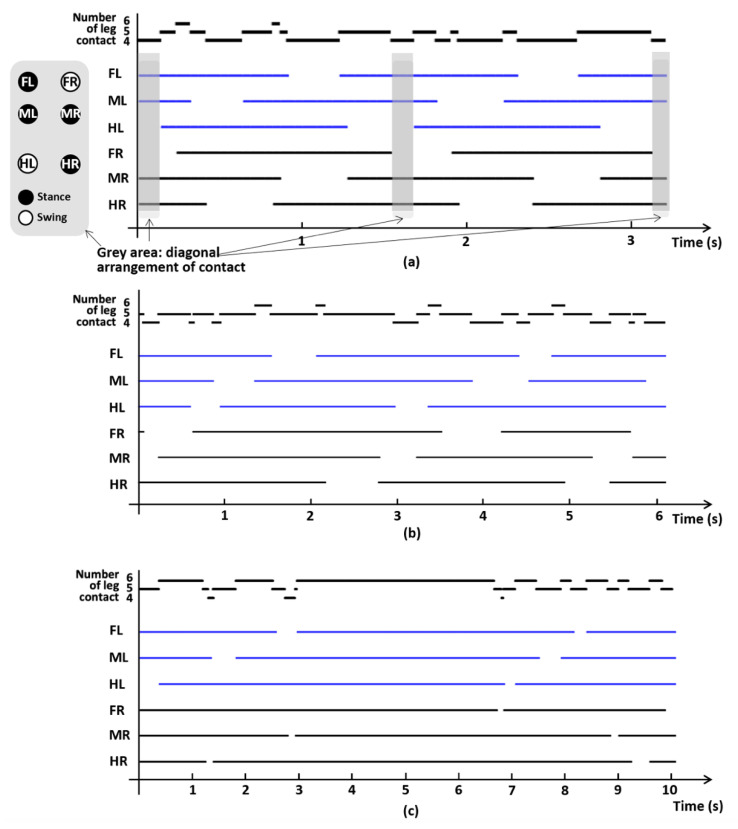
Example gait data from one of the three grasshoppers, representing stance or swing phase for each of six legs, at (**a**) 1.1, (**b**) 0.67, and (**c**) 0.21 cm/s. The solid line indicates stance phase and no line indicates swing phase. Each of the graphs represents fast, medium, and slow walking speeds. The grey areas in (**a**) indicate that overall legs on the ground are diagonally arranged, while this diagonal arrangement of contact cannot be found in other speed settings. FL: foreleg left-side, ML: midleg left-side, HL: hindleg left-side, FR: foreleg right-side, MR: midleg right-side, HR: hindleg right-side.

We quantified the temporal change in gait phase (stance or swing) of each leg at fast (1.1 cm/s), medium (0.67 cm/s), and slow (0.21 cm/s) walking speeds during two full cycles of the gait pattern to determine the exemplary phase relationship. This phase diagram shows that the mid-footfall follows hind-footfall and fore-footfall follows the mid-footfall. This walking sequence is similar to the lateral-sequence walking of the mammals, where the fore-footfall follows the hind-footfall. We also quantified the number of legs contacting the ground; the smallest number of contact legs was four, and at least two of three legs in each side maintained contact with the ground (Figure 8). The grey areas in Figure 8a indicate that overall limbs on the ground are diagonally arranged, while this diagonal arrangement of contact cannot be found in other speed settings. 

### 3.3. Number of Foot Contacts According to the Walking Speed

**Figure 9:**
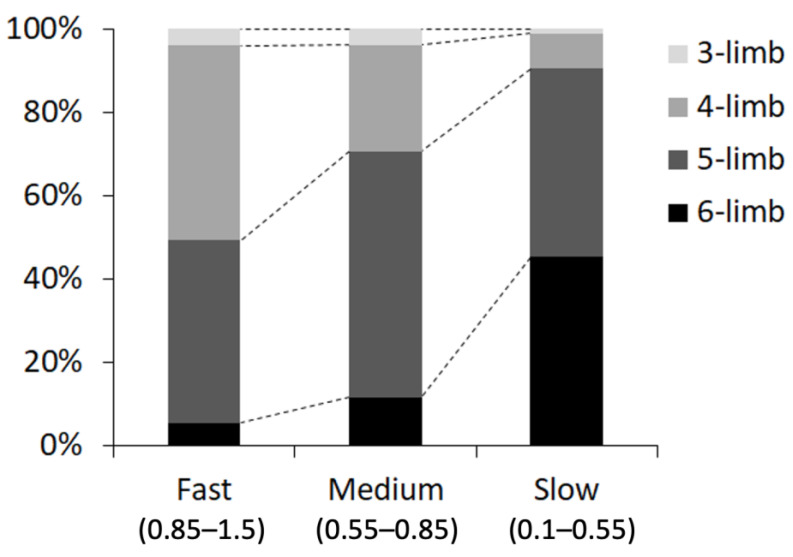
Proportion of each mode of leg contact (number of contact legs) at each walking speed: fast (0.85–1.5 cm/s), medium (0.55–0.85 cm/s), and slow (0.1–0.55 cm/s).

We also measured the percentage of the number of contact legs (three to six legs), at three ranges of walking speed: fast (0.85–1.5 cm/s), medium (0.55–0.85 cm/s), and slow (0.1–0.55 cm/s). We found that the grasshoppers mostly used four or five legs at a fast walking speed, five legs at a medium speed, and five or six legs at a slow speed (Figure 9). 

## 4. Discussion

This study represents the first application of a motorized treadmill to characterize insect gaits. Previous studies on insect gaits have relied on Styrofoam-based spherical treadmills, in which a test subject is usually glued dorsally on the thorax and allowed to walk on a light-weight sphere [33,48,49]. Although such systems, often coupled with a high-speed camera, can reveal important aspects of insect locomotion, they have some inherent limitations. For example, it is difficult to control and manipulate walking speed in these systems, as the treadmill simply provides a surface for a test subject to walk on. The types of insects that can be studied are also limited to small-bodied insects, such as ants, flies, and crickets, which are amenable to dorsal fixation. Therefore, the motorized treadmill system coupled with optical recording developed in our study allows a new opportunity to study insect gaits in a comparable manner to what has been applied to human and vertebrate gait research.

In order to demonstrate the utility of our new treadmill system, we deliberately selected a large, flightless, and slow-moving insect species as our test subject. We used the eastern lubber grasshopper, which was particularly suitable for our experiments for the following reasons: It is a large-bodied insect (~10 cm in body length) with short wings incapable of flight, and thus its primary mode of locomotion is walking. Although its hind legs are modified for jumping, we observed that jumping was used mostly as an escape behavior, and the grasshopper would normally walk slowly without jumping. Because of its large body and outward-facing legs, it was easy to place the reflective markers for optical recording. This species is easy to rear and maintain, as it has been frequently used in other biological research [50,51]. The biology and anatomy of the eastern lubber grasshopper are understood very well [52], and it is frequently used as an insect model for dissection in university-level introductory biology courses in the United States, which makes it easily accessible as a study organism. While the grasshoppers would readily walk on a regular surface, we did encounter some difficulties in making them walk on the motorized treadmill. After many trials and errors, we found several ways to help the training of the grasshoppers. First, we found that starvation would motivate the test subjects to walk on the treadmill, especially when a food reward was presented in front of them. The effectiveness of this method appeared similar to the approach employed in vertebrate models [53]. Second, we found that restraining the insect by fixating dorsally on the thorax, which is a standard method for the spherical treadmill system [33,48,49], did not work well for the motorized treadmill. We initially tried to forcibly place the grasshopper on a fixed location on the treadmill by attaching a rod on the top of their thorax using beeswax. Although the rod had a spring incorporated so that the rod was not pushing the grasshopper down, this method did not work well as the grasshopper simply refused to walk when fixed on the motorized treadmill. Instead, we found that the grasshopper would walk well on the motorized treadmill when completely unconstrained without any fixture. We simply provided two side walls at each side of the treadmill so that the grasshopper would not fall sideways, and this way, the grasshopper would behave in a way that could be used for measuring gaits. However, even with all the above conditions provided, many individuals would not walk consistently on the treadmill. Therefore, to collect a large amount of data with many replicates, further optimization is needed to improve the success rate. Nevertheless, our study represents a solid proof-of-concept that such a system can be adopted for large-bodied insects.

We also found some drawbacks of the current system that can be improved for future studies. First, the current gait analysis was based on the feature tracking algorithm with visual inspection of marker tracking yet did not quantify the error of the marker tracking algorithm. We will include a quantifiable error analysis for future study with a large amount of data. Second, the current system utilized a single camera for real-time gait analysis. Tian et al. [54] estimated parallax error ranges between 10 and 50% based on a single camera setup. Although we set the location of the camera about 80-85° from the ground to minimize the parallax error using a single camera, there can be a certain degree of parallax error involved. We plan to estimate the parallax error and minimize the error for the accurate distance measurement for better gait analysis and plan to add an additional camera to minimize the parallax error in our future study. 

Using this new motorized treadmill system with optical recording, we were able to characterize grasshopper gaits at different speeds and compare them with what is known from the vertebrate models [55,56]. 

The results of this study suggest that grasshoppers would be a valuable model in gait studies unveiling neural organization in the spinal cord for neuroscience and rehabilitation studies. Grasshoppers can provide a large number of samples for the study in a short time period with a high rate of reproduction and a short life cycle, and they have strong legs that are not easily broken during the gait study. Their anatomy and nervous system are understood well enough for us to interpret the experimental results [57]. In the follow-up study, we will measure kinetic parameters, such as contact force, and electromyogram for a complete and multifaceted gait analysis, as well as increasing the number of samples to confirm the findings.

## 5. Conclusions

Currently available gait analysis systems for insects are limited in controlling the walking speed and analyzing the gait of large-bodied insects in real-time. To overcome these limitations, we have developed a motorized treadmill with an optical recording system to analyze the gait of the large-bodied grasshoppers. As a feasibility study, we controlled the speed of the treadmill, collected the optical recordings of three grasshoppers, and analyzed the gait performance offline. From the output of the analysis, we found that the stance duty factor was negatively correlated with the treadmill speed. We also quantified the number of legs contacting the ground and learned the ratio of the number of contact legs changes with the treadmill speed. Even though the current study did not fully integrate the algorithm in real time, and we only collected walking data from three grasshoppers, this pilot study of the gait analysis of grasshoppers using the motorized treadmill with an optical recording system demonstrates the feasibility of gait analysis. We plan to include a greater number of grasshoppers with real-time data analysis in our future study. 

## Data Availability

Not applicable.
